# Nicotine Increases Spontaneous Glutamate Release in the Rostromedial Tegmental Nucleus

**DOI:** 10.3389/fnins.2020.604583

**Published:** 2021-01-13

**Authors:** Diego Castillo-Rolón, Enrique Ramírez-Sánchez, Gabina Arenas-López, Julieta Garduño, Omar Hernández-González, Stefan Mihailescu, Salvador Hernández-López

**Affiliations:** Departamento de Fisiología, Facultad de Medicina, Universidad Nacional Autónoma de México, Ciudad de México, Mexico

**Keywords:** nicotine, glutamate, synaptic release, calcium imaging, neuronal excitability

## Abstract

The rostromedial tegmental nucleus (RMTg) is a bilateral structure localized in the brainstem and comprise of mainly GABAergic neurons. One of the main functions of the RMTg is to regulate the activity of dopamine neurons of the mesoaccumbens pathway. Therefore, the RMTg has been proposed as a modulator of the reward system and adaptive behaviors associated to reward learning. The RMTg receives an important glutamatergic input from the lateral habenula. Also, it receives cholinergic inputs from the laterodorsal and pedunculopontine tegmental nuclei. Previously, it was reported that nicotine increases glutamate release, evoked by electric stimulation, in the RMTg nucleus. However, the mechanisms by which nicotine induces this effect were not explored. In the present work, we performed electrophysiological experiments in brainstem slices to study the effect of nicotine on spontaneous excitatory postsynaptic currents recorded from immunocytochemically identified RMTg neurons. Also, we used calcium imaging techniques to explore the effects of nicotine on multiple RMTg neurons simultaneously. We found that nicotine promotes the persistent release of glutamate through the activation of α7 nicotinic acetylcholine receptors present on glutamatergic afferents and by a mechanism involving calcium release from intracellular stores. Through these mechanisms, nicotine increases the excitability and synchronizes the activity of RMTg neurons. Our results suggest that the RMTg nucleus mediates the noxious effects of the nicotine, and it could be a potential therapeutic target against tobacco addiction.

## Introduction

The rostromedial tegmental nucleus (RMTg) is a bilateral mesencephalic structure localized posterior to the ventral tegmental area (VTA) and dorsal to the interpeduncular nucleus (IPN) (Perrotti et al., 2005; Jhou et al., 2009b; Sanchez-Catalan et al., 2014). The RMTg receives an important glutamatergic input from the lateral habenula (Jhou et al., 2009b; Kaufling et al., 2009; Lavezzi and Zahm, 2011), as well as cholinergic inputs from the laterodorsal (LDTg) and pedunculopontine (PPTg) tegmental nuclei (Yeomans, 2012; Wasserman et al., 2013; Yetnikoff et al., 2014). The RMTg has projections to the VTA, substantia nigra compacta (SNc), and the hypothalamus, among other structures (Jhou et al., 2009a,b; Hong et al., 2011; Lavezzi and Zahm, 2011; Bourdy and Barrot, 2012). Most of the RMTg projection neurons (≈72%) synthesize and release γ-aminobutyric acid (GABA) (Perrotti et al., 2005; Kaufling et al., 2010; Gonçalves et al., 2012). These GABAergic neurons have been shown to express specific transcription factors including Sox14, Sox2, and FoxP1 (Lahti et al., 2016). Among them, FoxP1 has been characterized as a reliable neurochemical marker for RMTg neurons in both rats and mice (Lahti et al., 2016; Smith et al., 2018). RMTg neurons projecting to the VTA and SNc also exhibit dense immunoreactivity to μ-opioid and somatostatin receptors (Jhou et al., 2009b). Because of its GABAergic influence, the RMTg exerts inhibitory control over mesolimbic dopamine systems (Jhou et al., 2009b; Hong et al., 2011; Kaufling and Aston-Jones, 2015), and it seems to play a role in reward processes, learning by reinforcement, and motivation behavior (Jhou et al., 2009a; Barrot et al., 2012; Bourdy and Barrot, 2012; Vento et al., 2017).

Several studies have shown that the activation of the RMTg produces aversion and avoidance-like behaviors to stimuli that are normally innocuous. On the opposite, the bilateral lesion of RMTg inhibits anxiety and adaptive behaviors to threatening or harmful stimuli (Jhou, 2005; Jhou et al., 2009a; Stamatakis and Stuber, 2012). Dysfunction of this nucleus could be associated with diverse pathologies such as anxiety and drug addictions (Jhou et al., 2013; Glover et al., 2019). Nicotine is one of the drugs of abuse most commonly used whose effects in the RMTg have been studied. It has been reported that nicotine increased the amplitude of excitatory postsynaptic currents (EPSCs) evoked by electrical stimuli and that this effect was blocked by an α7 nicotinic acetylcholine receptor (nAChR) antagonist (Lecca et al., 2012). However, the effect on spontaneous glutamate release and the action mechanisms of nicotine have not been explored with detail in this nucleus. In the present work, we studied the effects of nicotine on spontaneous glutamate release by measuring spontaneous excitatory postsynaptic currents (sEPSCs) recorded from identified RMTg GABAergic neurons, and we determined the calcium mechanisms involved in those effects. By using calcium imaging, we also studied the effect of nicotine on the excitability of RMTg neurons. We found that nicotine increases the frequency and the amplitude of sEPSCs through the activation of presynaptic α7 but not α4β2 nAChRs at glutamatergic terminals. These effects were dependent on calcium release from intracellular stores. Local application of acetylcholine (ACh) on RMTg neurons failed to produce nicotinic receptor–mediated currents discarding the presence of postsynaptic nAChRs. Our experiments also showed that nicotine promotes long-lasting glutamate release and synchronized firing of RMTg neurons. Our data confirm that nicotine activates the aversive system represented by the lateral habenula LHb and the RMTg nucleus.

## Materials and Methods

### Slices Preparation

All experiments were carried out following the National Institutes of Health Guide for the Care and Use of Laboratory Animals and were approved by the Institutional Animal Care Committee of the Universidad Nacional Autónoma de México. The experiments were performed in young male Wistar rats (25–30 postnatal days). Animals were anesthetized intraperitoneally with ketamine-xylazine (85–15 mg/kg) and then decapitated. Their brains were quickly removed and placed into ice-cold (4°C) artificial cerebrospinal fluid (ACSF) consisting of (in mM) 125 NaCl, 3 KCl, 25 NaHCO_3_, 1.25 NaH_2_PO_4_, 1 MgCl_2_, 1.2 CaCl_2_, and 25 glucose, 300 mOsm/L, pH 7.3 by bubbling with 95% O_2_–5% CO_2_. Coronal midbrain slices (250 μm thick) containing the RMTg nucleus were obtained with a vibratome (Pelco 102; Ted Pella, Inc) and stored in oxygenated ACSF at room temperature for at least 1 h before recordings.

### Whole-Cell Recordings

Individual slices were transferred into a custom-made Plexiglas recording chamber and superfused with ACSF at a rate of 4–5 mL/min. The temperature was maintained at 33°C with an in-line solution heater (TC-324B; Warner Instruments). RMTg GABAergic neurons were visualized by using an infrared video microscopy system (BX51WI; Olympus Instruments, Japan) fitted with an 80 × water-immersion objective. Whole-cell current-clamp and voltage-clamp recordings were performed with a Multiclamp 700B amplifier (Molecular Devices, LLC). Data were digitized by using a Digidata 1440A analog to digital converter (Molecular Devices, LLC) at a sampling rate of 5 kHz and stored in a personal computer running Clampex 10 software (Molecular Devices, LLC). Only one cell was recorded per brain slice. The recording electrodes were made from borosilicate glass tubes (WPI, Sarasota, FL) with a Flaming-Brown puller (Sutter Instruments, Novato, CA). The impedance of the electrodes varied between 4 and 7 MΩ. The internal solution consisted of (in mM) 140 K-gluconate, 5 NaCl, 1 MgCl_2_, 0.02 EGTA, 10 HEPES, 2 Mg_2_-ATP, 0.5 Na_2_GTP, and 0.3% biocytin, pH 7.2–7.3, with Trizma base, 280–300 mOsm/L. In an experimental group, we added 1-2-bis(2-aminophenoxy)ethane-N,N,N,N-tetraacetic acid (BAPTA) (10 mM) in the same internal solution with 130 mM K-gluconate (Figure 5A). Each recorded cell was first identified based on its electrophysiological characteristics by generating a stimulus–response relationship in current-clamp mode. sEPSCs were recorded continuously in whole-cell voltage-clamp mode at a holding potential of -70 mV. All experiments were made in the presence of gabazine or bicuculline (10 μM) to block GABA_*A*_ receptors. Access resistance was monitored by using depolarizing step pulses of 5 mV and 50 ms duration. When access resistance varied > 15%, the experiment was discarded. One limitation in our experiments is the space clamping because in slice recordings it is impossible to reach a good voltage clamping in cell areas remote from the soma, for instance, the dendrites. However, the RMTg neurons have a small average size (≈17 μm diameter), which allows a good voltage clamping in the soma, and in general, the recordings of sEPSCs were stable during the experiments.

### Calcium Imaging

These methods have been described before (Carrillo-Reid et al., 2008; Garduño et al., 2019). Briefly, slices containing the RMTg nucleus were incubated in the dark for 25–30 min at 27°C with 10 μM of fluo 4 AM (Invitrogen, Life Technologies) in 0.1% dimethyl sulfoxide and dissolved in the same ACSF solution. The cells were visualized by using an Eclipse FN-1 microscope (Nikon) equipped with a 16 × water-immersion objective. The image field visualized was 280 × 260 μm size. The fluorophore was stimulated with light pulses at 488-nm wavelength (15—25 ms exposure time) delivered with a Lambda HPX-L5 lamp (Sutter Instruments) connected to the microscope by optic fiber. Image sequences or videos were acquired at a frequency of 4 frames/s (250 ms/frame) by using a digital camera (Cool SNAP MYO, Photometrics) and a custom-made software designed in the LabView environment (Im-Patch) (Pérez-Ortega et al., 2016). Videos lasted 6 min (1,440 frames). Active cells were selected in each video by using a circular template of variable size (4–30 μm), and a coordinates map of active neurons was automatically generated from the summary image by using Im-Patch software (Pérez-Ortega et al., 2016). The intracellular calcium signals (calcium transients) from each region of interest were collected over time. Calcium-dependent fluorescence signals were computed as (Δ*F*/*F*), where Δ*F* is fluorescence intensity at any frame, and *F* is resting fluorescence. Calcium transients and their first derivative were taken as indicators of cell firing (Carrillo-Reid et al., 2008). Only transient events with amplitudes 2.5 times above the standard deviation of the noise were considered for analysis. From calcium transients, a binary matrix of ones and zeros was built for each experiment, in which each row belongs to an active cell and each column to a frame, where 1 indicates an active and 0 an inactive frame for each neuron. The *y*-axis in the matrix represents the number of active neurons, and the *x*-axis represents the number of image frames in a video. Matrices were used to generate raster plot graphs that illustrated the activity of all the cells along with the experiment. The summed activity was graphed below the raster plot in a form of a histogram (coactivity histogram) (Carrillo-Reid et al., 2008). Monte Carlo simulations (1,000) were used to find the significance of neurons being active together (Carrillo-Reid et al., 2008, 2011; Pérez-Ortega et al., 2016; Lara-González et al., 2019). MATLAB was used to analyze the activity of individual cells along with the different conditions as described in Aparicio-Juárez et al. (2019).

### Drug Administration

All drugs were dissolved into the ACSF from daily made stock solutions and administered at a 4 mL/min speed by using a gravity-driven superfusion system. A complete experiment consisted of three stages: a control recording period, the drug administration, and an additional recording period after the drug washout. Each of these stages was taken for analysis. The control recordings consisted of 10–15 min to allow the stabilization of sEPSC frequency. This control was taken in the presence of GABA_*A*_ receptors blockers (gabazine or bicuculline). In the drug administration stage, nicotine, eserine, or nAChRs agonists were added to the perfusion fluid for 8–10 min. After the drug washout, an additional recording period of at least 30 min was taken. In those experiments where nAChRs antagonists, tetrodotoxin (TTX), or calcium-channel blockers were used, the drug was administered for at least 10 min, and its effects on sEPSCs were recorded. Afterward, nicotine was added to the perfusion fluid for 8–10 min, and a final 30–40 min recording period was taken after nicotine washout. TTX, atropine, 6-cyano-2,3-dihydroxy-7-nitroquinoxaline (CNQX), SR-95531 (gabazine), (-)bicuculline methiodide, methyllycaconitine (MLA), dihydro-β-erytroidine hydrobromide (DHβE), BAPTA, BAPTA-AM, cadmium chloride (CdCl_2_), and biocytin were purchased from Sigma–Aldrich/RBI (St Louis, MO). Thapsigargin, ryanodine, cyclopiazonic acid (CPA), (E)-N-methyl-4-(3-pyridinyl)-3-buten-1-amine oxalate (RJR-2403 oxalate), and N-(3R)-1-azabicyclo[2.2.2]oct-3-yl-4-chlorobenzamide (PNU-282987) were purchased from Tocris Bioscience (Ellisville, MO).

### Local Administration of ACh

Local applications of ACh (1 mM) on RMTg or the VTA neurons were made by using a microinjector (IM 300, Narisihige Comp., Japan) coupled to a fine-tip glass micropipette placed about 50 μm above the recorded neuron. Brief “puffs” of ACh (2–5 psi, during 500 ms) were applied in the presence of atropine (5 μM) and TTX (1 μM) to eliminate the muscarinic component of the response and avoid indirect effects (Galindo-Charles et al., 2008). Intervals of 3–5 min were left between each ACh administration to avoid desensitization of the nicotinic receptors (Wooltorton et al., 2003).

### Immunocytochemistry

A combination of intracellular labeling and GAD_65/67_ or FoxP1 immunocytochemistry was used to identify RMTg recorded neurons. Neurons were filled with biocytin dissolved in the internal solution. At the end of the experiment, the slices containing recorded neurons were fixed for 48 h with 4% paraformaldehyde in 0.1 M phosphate-buffered saline (PBS) (pH 7.4). The slices were then washed twice with PBS, put into a 30% sucrose solution at 4°C overnight, and cut into coronal sections (40 μm thick) with a vibratome. Then, the sections were incubated for 24 h in a PBS solution containing 0.25% Triton X-100 and streptavidin conjugated to Cy3 (Invitrogen, Carlsbad, CA, dilution 1:200) to label the recorded cell. The sections were rinsed in PBS and incubated for 18–24 h with primary antibodies. We used the anti-GAD_65/67_ antibody produced in rabbit (Sigma–Aldrich, dilution 1:1,000) and rabbit anti-FoxP1 (Sigma–Aldrich, dilution 1:1,000) in blocking solution. Afterward, sections were washed three times with PBS and reincubated with a secondary antibody conjugated to fluorescein (Vector Laboratories, Burlingame, CA, dilution 1:200) for 30 min at room temperature. Reacted sections were washed three times for 10 min in PBS, mounted in an antiquenching medium (Vectashield, Vector Laboratories), and examined under a confocal microscope (MRC 1024, Bio-Rad, Natford, United Kingdom) equipped with a krypton/argon laser. A two-line laser emitting at 550 and 500 nm wavelength was used for exciting Cy3 and fluorescein, respectively. Digitized images were transferred to a personal computer by using the image-capturing software (Confocal Assistant, T. C. Brelje, Minneapolis, MN). The omission of primary antisera resulted in no detectable signal (data not shown).

### Data Analysis

Offline analysis of the data was performed using Clampfit 10.2 (Molecular Devices), Mini Analysis (Synaptosoft, Decatur, GA), and graphing and statistical software packages (Origin 8, OriginLab, Northampton, MA; and GraphPad Prism 6, San Jose CA, United States). MiniAnalysis software was used to detect and analyze sEPSCs. Initially, a noise analysis was conducted for each recorded cell, and detection thresholds were set to exceed noise values. For each recorded cell, 10 s bin sEPSCs or mEPSC frequency histograms were constructed, and then all the cells of each experimental group were averaged. The changes in sEPSC frequency or amplitude produced by drug administration were expressed as values normalized to the baseline (“control”) for each experimental group. We took 5 min of the maximal effect as the window of analysis for statistical comparisons. Upon small samples, we used the Mann–Whitney *U* and Friedman tests. To compare distributions, we used the Kolgomorov–Smirnov test. The data are expressed as means ± SEM. For each experimental group, a minimum of five cells was recorded. *P* < 0.05 was taken as statistically significant.

## Results

We recorded 121 RMTg cells, which were first identified as being GABAergic neurons based on their electrophysiological properties: high spontaneous firing rate (≈17 Hz), a biphasic and brief action potential (duration < 1.5 ms), and short after-hyperpolarizing-potential duration (Jhou et al., 2009b; Lecca et al., 2011). During the recording experiments, RMTg neurons were filled with biocytin dissolved in the internal solution. After the experiments, they were identified by immunocytochemistry by using anti-GAD_65/67_ or anti-FoxP1, two of the main markers of the RMTg neurons (Jhou et al., 2009b; Lahti et al., 2016; Smith et al., 2018). Eighty of 121 cells were immunocytochemically identified. From these neurons, 33 were FoxP1-positive, and 47 were GAD-positive. The data reported here are from all the responsive cells independently if they were immunocytochemically identified or not. A cell was taken as responsive when there was a statistically significant change, with respect to the baseline, within the 10 min after the drug administration.

### Nicotine Increased the Frequency and Amplitude of sEPSCs Recorded From GABAergic RMTg Neurons

**FIGURE 1:**
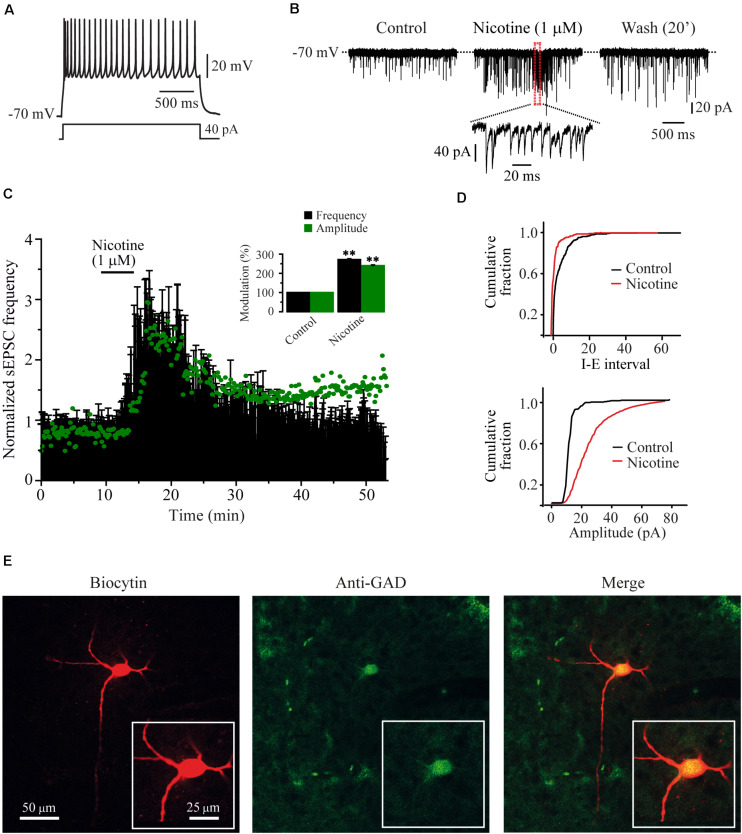
Nicotine increased the frequency and evoked bursts of sEPSCs recorded from RMTg GABAergic neurons. **(A)** Voltage response of a typical GABAergic RMTg neuron to a depolarizing current step of 40 pA. **(B)** Current traces showing sEPSCs recorded in voltage clamp mode from the same cell in A. Control condition (left), 10 min after nicotine (middle) and 20 min after nicotine washout (right). A region of the middle trace (red box) is displayed below at a slower sweep time where nicotine-evoked bursts of sEPSCs are evident. **(C)** Time-frequency histogram (*n* = 15) shows the temporal course of nicotine effect. The graph in green shows the time course of the sEPSC amplitude. For clarity, the errors were removed from the graph. The summary of the 15 cells is shown in the bar graph (inset) (Mann–Whitney *U*-test). **(D)** Cumulative fraction plots (cell in A,B) of the inter-event interval (I–E, top) and amplitude (bottom) showing that nicotine increased both the frequency and the amplitude of sEPSCs. **(E)** Same cell in A and B labeled with biocytin (left), anti-GAD_65/67_ antibody (middle), and merge (right). All the experiments were performed in the presence of bicuculline (10 μM). **p < 0.01.

The nicotine-evoked increases in frequency and amplitude of sEPSCs were observed in 15/19 GABA-positive recorded cells. RMTg neurons were identified based on their firing features (Figure 1A); sEPSCs were recorded in voltage-clamp mode from a holding potential of -70 mV (Figure 1B). All the experiments were made in the presence of bicuculline (10 μM). The bath application of nicotine (1 μM) increased the sEPSC frequency by 172% ± 3.9% with respect to the baseline (Mann–Whitney *U*-test, *p* = 0.0014, *n* = 15), and this effect persisted for about 25–30 min after the drug washout (Figures 1B–D, top). Besides the frequency, nicotine increased the amplitude of the sEPSCs. The time course of the average change in amplitude is shown in Figure 1C (green dots). The inset graph shows the frequency and amplitude increase. The amplitude increase was 140.3% ± 3.8% compared to the baseline. Figure 1D (bottom) shows the cumulative fraction of the sEPSC amplitudes for the cell in A and B (two-sample Kolgomorov–Smirnov test, *p* = 0.0001). The effect on the amplitude suggests a possible postsynaptic effect (Zucker and Regehr, 2002; Garduño et al., 2012). Previously, it was reported that nicotine can evoke bursts of sEPSCs. This bursting activity is important because it suggests a synchronized release of glutamate from the terminals (Sharma and Vijayaraghavan, 2003; Sharma et al., 2008). In this work, a burst was defined as a short period of time 50–150 ms, where the frequency of synaptic events exceeded 50% of the frequency observed in the baseline. We found that nicotine evoked bursts of sEPSCs in 93% of the recorded cells (Figure 1B inset). In six experiments, CNQX (10 μM), an antagonist of AMPA/kainate receptors, was applied after nicotine washout. In all the recorded cells, CNQX completely abolished sEPSCs, demonstrating their glutamatergic nature (not shown). The recordings shown in Figures 1A,B belong to the GAD_65/67_-positive cell illustrated in Figure 1E.

### Endogenous ACh Mimicked the Effects of Nicotine

**FIGURE 2:**
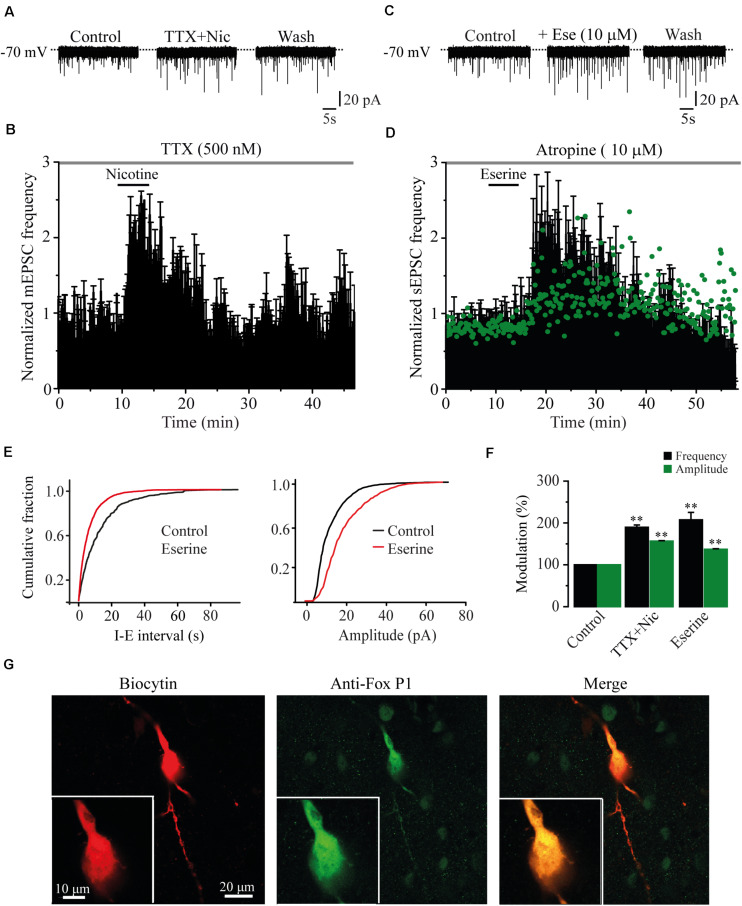
The effects of nicotine are not dependent of action potential. **(A)** Current traces showing sEPSCs in control condition (left), 10 min after nicotine plus TTX (500 nM, middle), and 20 min after nicotine washout (right). **(B)** Time-frequency histogram shows the temporal course of the effect of nicotine in the presence of TTX (*n* = 6). **(C)** Current traces showing sEPSCs in control condition (left); 10 min after eserine (10 μM, middle), an inhibitor of the enzyme acetylcholinesterase; and 20 min after eserine washout (right). **(D)** Time-frequency histogram shows the time course of the effect of eserine (*n* = 8). The graph in green, shows the time course of the amplitude. For clarity, the errors were removed from the graph. **(E)** Cumulative fraction plots (cell in C) of the inter-event interval (I–E, left) and amplitude (right) showing that eserine increased the frequency and amplitude of sEPSCs. **(F)** Summary of the results (Mann–Whitney *U*-test). **(G)** Same cell in A labeled with biocytin (left), anti-Fox P1 antibody (middle), and merge (right). Eserine experiments were performed in the presence of atropine (10 μM). **p < 0.01.

The action potential dependence of the nicotinic effect was tested in six cells by adding TTX (500 nM) to the bath. In the presence of TTX, nicotine (1 μM) still enhanced the frequency of mEPSCs (Figures 2A,B). mEPSC frequency was increased by 89.7% ± 5.6% with respect to the baseline (Mann–Whitney *U*-test, *p* = 0.0021, *n* = 6) and the amplitude (56.5% ± 1.2%) (Figure 2F and Supplementary Figure 1), indicating that the nicotinic effect is independent of the action potential. To investigate if endogenous ACh had the same effect as nicotine, we applied eserine (10 μM), an acetylcholinesterase inhibitor. Eserine increased the sEPSC frequency in eight RMTg recorded cells (Figures 2C–E left,F), and the effect was also persistent as shown for nicotine. sEPSC frequency increased by 107.4% ± 17.8% with respect to the baseline (Mann–Whitney *U*-test, *p* = 0.0016). The amplitude increase was 37% ± 1.5% (Figures 2D green dots and F), suggesting that endogenous ACh is tonically regulating glutamate release in the RMTg nucleus. Figure 2E (right) shows the increase in the sEPSC amplitude of the cell in C (two-sample Kolgomorov–Smirnov test, *p* = 0.001). The recording shown in Figure 2C belongs to a cell that was positive to FoxP1, another marker of RMTg neurons (Lahti et al., 2016; Figure 2G).

### The Effects of Nicotine Were Mediated Through α7 nAChRs

**FIGURE 3:**
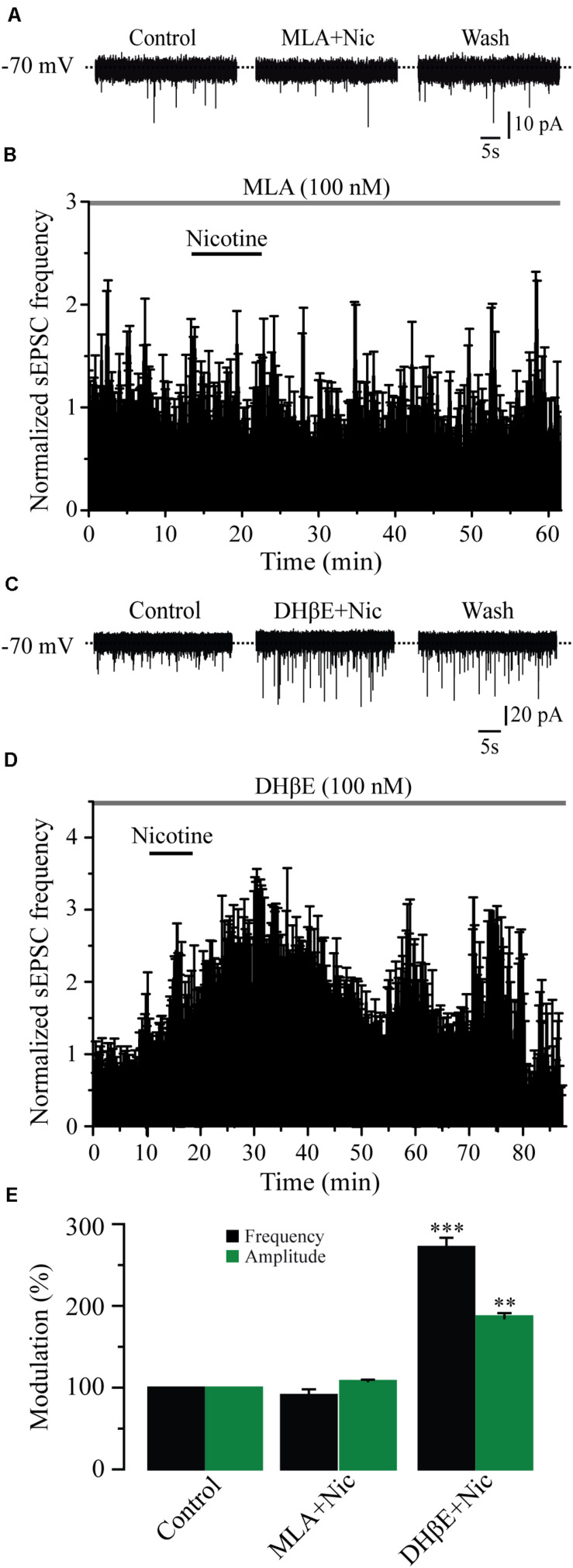
Nicotine effects are mediated through α7 but not α4β2 nAChRs. **(A)** Current traces showing that MLA (100 nM), a selective antagonist of the α7 nAChRs, completely blocked the effects of nicotine on sEPSCs, control condition (left), 10 min after nicotine plus MLA (middle), and nicotine washout (right). **(B)** Normalized time-frequency histogram showing the lack of effect of nicotine in the presence of MLA (*n* = 6). **(C)** Current traces showing that DHβE (100 nM), a selective antagonist of the α4β2 nAChRs did not block the effect of nicotine on sEPSCs. **(D)** Normalized time-frequency histogram showing that nicotine increased the frequency of sEPSCs in the presence of DHβE (*n* = 6). **(E)** Bar graph illustrating a summary of the data (Mann–Whitney *U*-test). **p < 0.01, ***p < 0.001.

**FIGURE 4:**
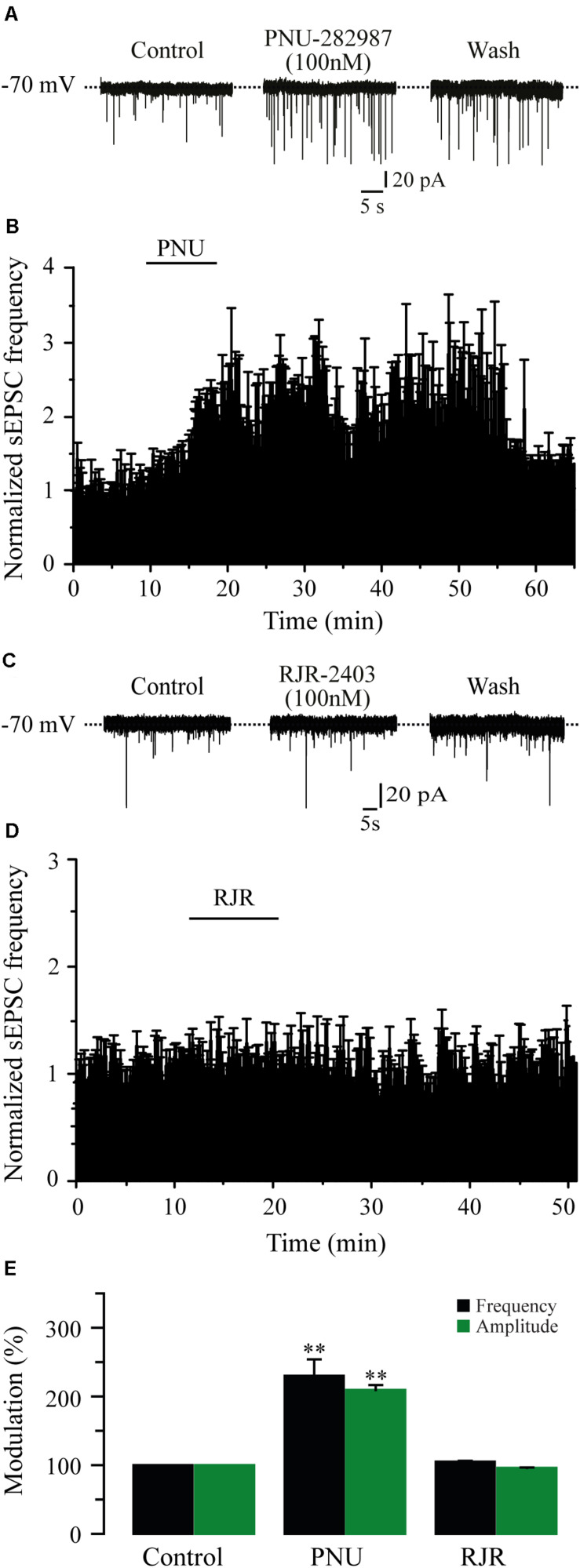
The activation of α7 nAChRs with a selective agonist mimicked the effect of nicotine. **(A)** Current traces showing sEPSCs in control condition (left), 10 min after the application of PNU-282987, a selective agonist of α7 nAChRs (PNU 100 nM, middle), and 20 min after PNU washout (right). **(B)** Time-frequency histogram showing the time course of PNU effects (*n* = 6). **(C)**, Current traces showing sEPSCs in control condition (left), 10 min after the application of RJR-2403, a selective agonist of the α4β2 nAChRs (RJR 100 nM, middle), and 20 min after RJR washout (right). **(D)** Normalized time-frequency histogram showing the lack of effect of RJR (*n* = 6). **(E)** Summary of the data (Mann–Whitney *U*-test). **p < 0.01.

It is well known that the α4β2 and the α7 subtypes are the most commonly expressed nAChRs in the CNS (Whiting and Lindstrom, 1986). To identify which subtype of nAChRs was mediating the effects of nicotine, we tested the selective nAChRs antagonists MLA and DHβE. Blocking the α7 nAChRs with MLA (100 nM) in six cells completely suppressed the effect of nicotine on sEPSC frequency (Figures 3A,B,E). At this concentration, MLA did not produce any significant effect by itself, but it blocked the nicotinic effects (91% ± 6.9% with respect to the baseline, Figure 3E) (Mann–Whitney *U*-test, *p* = 0.1752, *n* = 6). MLA also blocked the increase in sEPSC amplitude induced by nicotine (Figure 3E and Supplementary Figure 2A). In contrast, in the presence of DHβE (100 nM), an antagonist of the α4β2 nAChRs, nicotine increased both the frequency and amplitude of sEPSCs in six cells. The sEPSC frequency increased by 172% ± 11.2% with respect to the baseline, and the effect persisted for more than 30 min after nicotine washout (Figures 3C–E) (Mann–Whitney *U*-test, *p* = 0.001, *n* = 6). The amplitude increase was 86% ± 4.8% (Figure 3E and Supplementary Figure 2B). To further investigate the identity of the nAChR subtype involved in nicotine-induced glutamate release, we used α7 and α4β2 nAChRs-selective agonists. The selective α7 nAChR agonist, PNU-282987 (100 nM), mimicked the effect of nicotine as it increased the frequency in six cells (Figures 4A,B,E). The frequency increase produced by PNU was 130% ± 23.9% with respect to the baseline, and it was statistically significant (Mann–Whitney *U*-test, *p* = 0.0013, *n* = 6). The amplitude increase was 110.4% ± 5.6% (Figure 4E and Supplementary Figure 3A). Conversely, the selective α4β2 nAChR agonist RJR-2403 (100 nM) did not change the sEPSC frequency or amplitude in any of the six GABAergic-positive recorded cells (Figures 4C–E and Supplementary Figure 3B). These data suggest that nicotine regulates glutamate release by activating α7 but not α4β2 nAChRs in the glutamatergic terminals of the RMTg nucleus.

### Nicotine Increased EPSCs Frequency and Amplitude by Presynaptic Mechanisms

**FIGURE 5:**
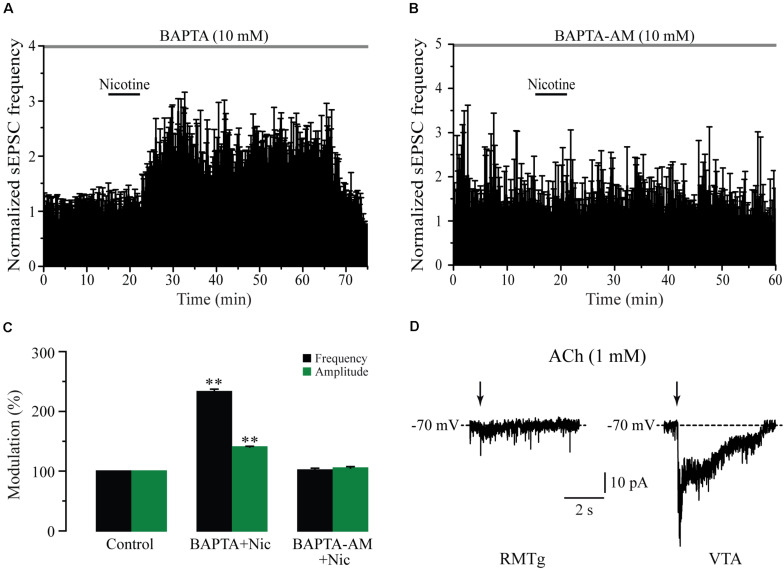
Nicotine action mechanisms are presynaptic. **(A)** Time-frequency histogram showing the time course of nicotine effect with BAPTA in the recording pipette (*n* = 5). **(B)** Time-frequency histogram showing the time course of nicotine effect in the presence of the membrane permeable BAPTA-AM (10 mM) added in the bath solution (*n* = 6). **(C)** Bar graph illustrating a summary of the data (Mann–Whitney *U*-test). **(D)** Voltage-clamp recordings of currents after local application of “puffs” of ACh (1 mM) onto a RMTg (left) or a VTA neuron (right). These experiments were made in the presence of atropine (5 μM) and TTX (500 nM). **p < 0.01.

As nicotine and the α7 nAChR agonist increased not only the frequency but also the amplitude of sEPSCs, it is possible that these drugs activated postsynaptic nAChRs. To test this possibility, we buffered the postsynaptic calcium by loading the patch pipette with an internal solution containing BAPTA (10 mM) in five cells. It was observed that intracellular BAPTA had no effect on spontaneous synaptic activity and did not prevent the effects of nicotine on sEPSCs. In these conditions, nicotine increased sEPSC frequency by 133% ± 4.2% with respect to the baseline (Figures 5A,C) (Mann–Whitney *U*-test, *p* = 0.0018, *n* = 5). As expected, sEPSC amplitude was also increased (40% ± 2%) (Figure 5C and Supplementary Figure 4A). On the other hand, nicotine did not have any effect on the frequency or the amplitude of sEPSCs when the slices were perfused for 30 min with the membrane-permeable calcium-chelator BAPTA-AM (10 mM) in six recorded cells (Figures 5B,C and Supplementary Figure 4B). To further discard the presence of postsynaptic nAChRs, we locally applied ACh onto 20 RMTg neurons recorded in voltage-clamp mode at a holding potential of -70 mV. Brief (500 ms) “puffs” of ACh (1 mM) were applied by using a glass micropipette located on top of the recorded cell (see section “Materials and Methods”). These experiments were performed in the presence of atropine (5 μM) and TTX (1 μM) to block muscarinic receptors and discard indirect effects, respectively. ACh did not evoke inward currents in any of the 20 RMTg recorded cells (Figure 5D, left). In contrast, in three of four VTA neurons, in which the presence of nAChRs was previously reported (Wooltorton et al., 2003), “puffs” of ACh evoked fast and slow inward currents (Figure 5D, right). The fast component exhibited a high amplitude and short decay time and is mediated by α7 nAChRs. The other component, with smaller amplitude and slower kinetic, corresponds to an inward current mediated by α4β2 nAChRs (Pidoplichko et al., 1997; Klink et al., 2001; Wooltorton et al., 2003; Wu et al., 2004). Taken together, these results support the idea that nicotinic effects are presynaptic and mediated through the activation of α7 nAChRs located at glutamate terminals in the RMTg nucleus.

### Nicotine-Induced Synaptic Potentiation Depends on Intracellular Calcium Stores

**FIGURE 6:**
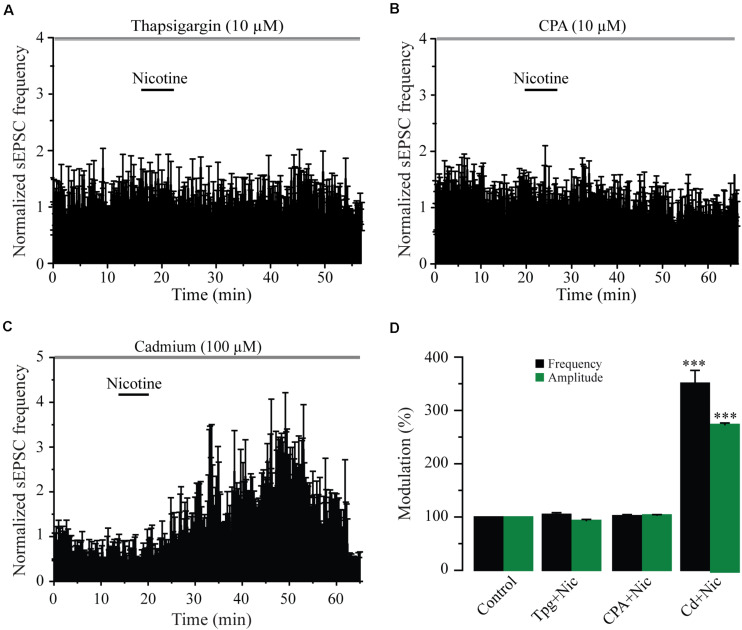
Blockage of intracellular calcium stores abolishes the effects of nicotine. **(A)** Time-frequency histogram showing the lack of effect of nicotine on the sEPSC frequency in the presence of the SERCA blocker thapsigargin (10 μM) (*n* = 6). **(B)** Time-frequency histogram showing the lack of effect of nicotine on the sEPSC frequency in the presence of cyclopiazonic acid (CPA, 10 μM) (*n* = 6). **(C)** Time-frequency histogram showing that nicotine had strong effects on sEPSCs in the presence of cadmium (100 μM), a blocker of the VGCCs (*n* = 6). **(D)** Bar graph illustrating a summary of the data (Mann–Whitney *U*-test). ***p < 0.001.

Previously, it has been shown that nicotine-induced glutamate release involves a calcium-induced calcium release (CICR) mechanism acting through either α7 (Sharma and Vijayaraghavan, 2003; Sharma et al., 2008) or β2 subunit-containing nAChRs (Dajas-Bailador et al., 2002; Dickinson et al., 2008; Garduño et al., 2012). To explore this issue, we used thapsigargin (six cells) and CPA (six cells), two blockers of the sarcoplasmic/endoplasmic reticulum calcium ATPase pump (SERCA). In the presence of thapsigargin (10 μM) or CPA (10 μM), nicotine did not change the sEPSC frequency or the amplitude with respect to the blocker alone, taken as the control (Figures 6A,B,D and Supplementary Figures 5A,B) (Mann–Whitney *U*-test, *p* = 0.1530, *n* = 6). We also explored the involvement of voltage-gated calcium channels (VGCCs) by testing the effect of nicotine in the presence of cadmium (CdCl_2_, 100 μM) in seven cells. In the presence of cadmium, nicotine still increased the sEPSC frequency in all the recorded cells by 251% ± 24% (Figures 6C,D) (Mann–Whitney *U*-test, *p* = 0.0001, *n* = 7), a value that is much higher when compared with the effect of nicotine alone. This could be explained by a potentiation of nAChRs produced by cadmium (Hsiao et al., 2001; Garduño et al., 2012). As observed before, nicotine also increased the sEPSC amplitude by 173.2% ± 4% with respect to the baseline (see Supplementary Figure 5C). These data indicate that nicotine-induced synaptic potentiation of glutamate release requires calcium from intracellular stores of glutamatergic afferents and does not depend on calcium influx through VGCCs.

### Nicotine Synchronized Glutamate Release in the RMTg Nucleus

**FIGURE 7:**
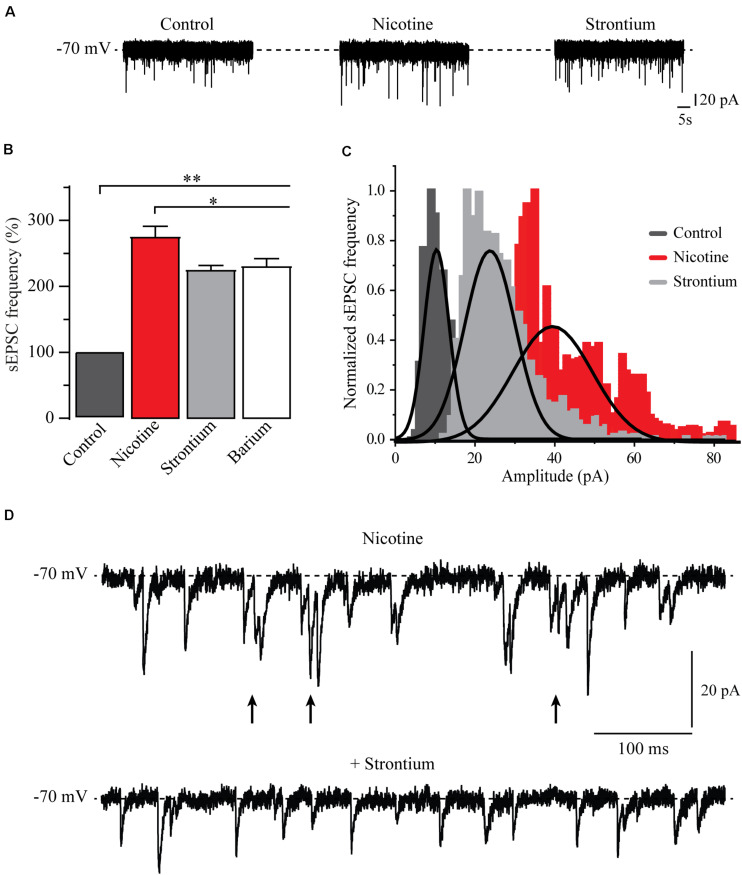
Nicotine synchronizes glutamate release. **(A)** Current traces showing sEPSCs in control condition (left), 10 min after the application of nicotine (middle), 10 min after replacing 90% of the extracellular calcium by strontium (right). **(B)** Bar graph of the sEPSC frequency that compares different conditions: control (dark gray) nicotine (red) strontium (light gray), and barium (white) (Mann–Whitney *U*-test). **(C)** Amplitude histograms for control (dark gray), nicotine (red), and strontium (light gray). **(D)** Current trace showing a typical sEPSC bursting evoked by nicotine (top). Note that the addition of strontium abolished the bursting and decreased the amplitude of sEPSCs (bottom). *p < 0.05, **p < 0.01.

Previously, it was reported that nicotine may synchronize the release of glutamate in other cells (Sharma and Vijayaraghavan, 2003; Sharma et al., 2008). To test this idea, we replaced 90% of calcium with the divalent cations, strontium (6 cells), or barium (6 cells) in the external solution to desynchronize synaptic release (Léna and Changeux, 1997; Xu-Friedman and Regehr, 2000; Good and Lupica, 2009). Immediately after the nicotine effect was patent, the normal calcium external solution was substituted by a strontium external solution (see section “Materials and Methods”). As expected, for a normal external solution, nicotine increased the frequency and amplitude of sEPSCs. However, when strontium was added, a decrease in the amplitude of sEPSCs was noticed (Figure 7A). The bar graph (Figure 7B), shows the sEPSC frequency normalized to the control. Clearly, nicotine and the divalent cations increased the sEPSC frequency with respect to the control. The frequency increments were as follows: 176% ± 14%, 139% ± 6% (*n* = 6), and 152% ± 10% (*n* = 6), for nicotine, strontium, and barium, respectively (Mann–Whitney *U*-test, *p* = 0.0017). Besides, it was observed that strontium and barium produced a small but statistically significant reduction of the sEPSC frequency when compared to nicotine (Mann–Whitney *U*-test, *p* = 0.035, *n* = 6). Figure 7C illustrates an amplitude histogram where the control (dark gray) and the nicotine (red) represent the amplitude distributions in normal calcium external solution. Nicotine shifted the distribution of the amplitudes to the right. When calcium was replaced with strontium (light gray), the amplitude distribution was shifted to the left, toward the control. sEPSC amplitude increments, with respect to the baseline, were 139.7% ± 2.4%, 107.7% ± 1.7% (*n* = 6), and 89.9% ± 1.7% (*n* = 6) for nicotine, strontium, and barium, respectively. Nicotine was statistically different from both strontium and barium (two-sample Kolmogorov–Smirnov test, *p* = 0.047, *n* = 6). Figure 7D shows current traces of an individual neuron after 10 min of nicotine application (top) followed by strontium (bottom). As previously shown, nicotine not only increased the frequency and the amplitude but also caused bursts of sEPSCs. Figure 7D (top) shows bursting activity (arrows) with a frequency of about 47 Hz compared to the control frequency before nicotine, which was 3.5 Hz (not shown). It was observed that after adding the strontium external solution, the bursting activity was abolished (Figure 7D bottom) while the frequency remained high.

### Nicotine Increased the Activity of RMTg Neurons, and the Effect Was Blocked by MLA

**FIGURE 8:**
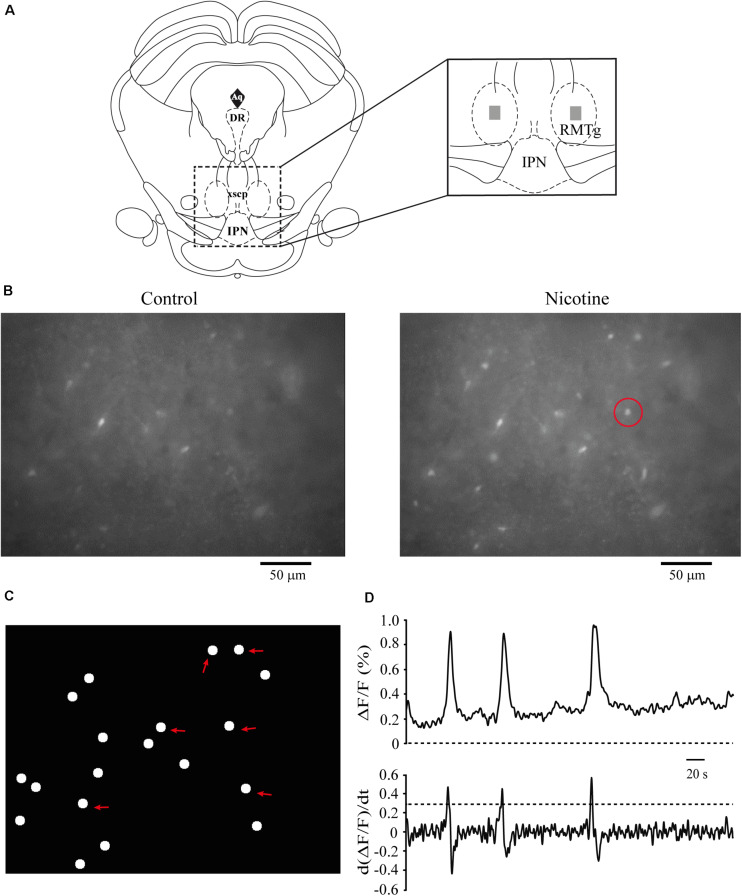
Calcium imaging recordings. **(A)** Schematic representation of a coronal slice containing the RMTg area. The zone within the square was amplified (right). The dark gray rectangles indicate the region within the RMTg where calcium image recordings were made. **(B)** Raw data of calcium imaging recordings in control conditions (left) and nicotine (1 μM, right). Each image was obtained from a single frame of the video. **(C)** Scheme showing the coordinates map of the active cells constructed from the video (see section “Materials and Methods”). The red arrows indicate the locations of the cells recorded in **(B)**. **(D)** Calcium transients (top) and their first derivate (bottom), which correspond to the cell encircled in **(B)** (right, red circle). RMTg, rostromedial tegmental nucleus; IPN, interpeduncular nucleus.

**FIGURE 9:**
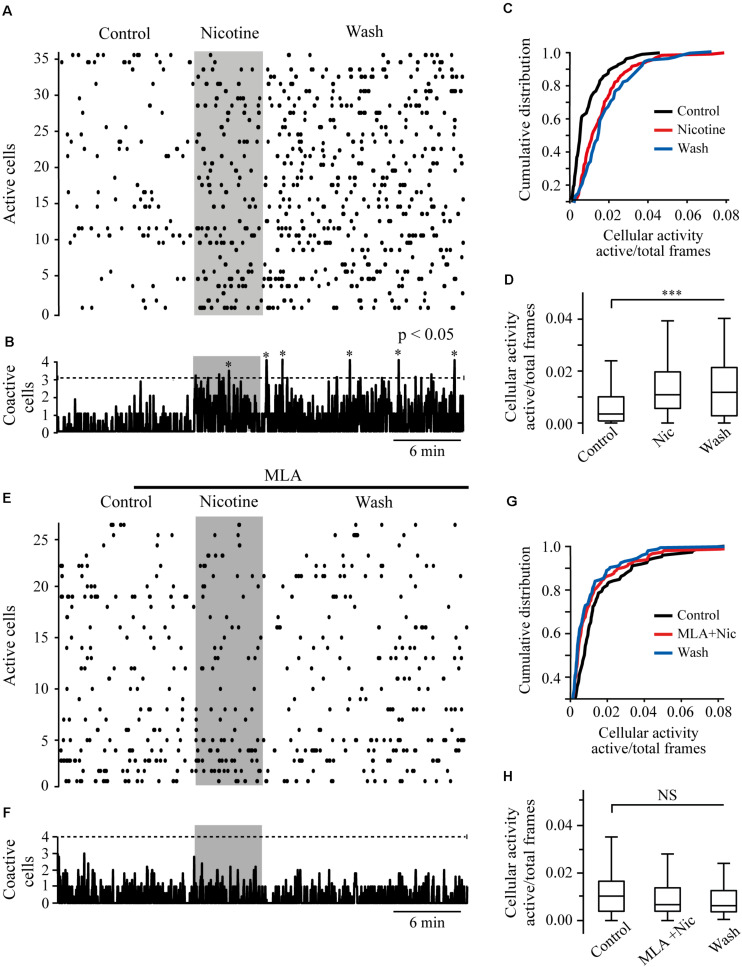
Nicotine increases RMTg neuronal activity. **(A)** Raster plot showing the spontaneous activity of 36 cells from a single experiment in RMTg nucleus. Rows represent the activity of individual neurons during the total time of the experiment. Columns are video frames converted to time in minutes. Note that cell activity is increased in nicotine (gray band) and wash conditions as compared with control. **(B)** Histogram showing coactive cell peaks, which represent the number of neurons that fired together in a given frame. Those peaks (asterisks) are statistically significant when they overpass the threshold (Monte Carlo test). Note that coactivity peaks occurrence started in nicotine but were more evident during the wash time. **(C)** Cumulative distribution of cellular activity of all cells in the three different conditions. Note that in nicotine (red line) and wash (blue line) conditions, the distributions were shifted to the right with respect to the control (black line), which indicates greater cellular activity. The distributions of nicotine and wash were statistically different as compared to control (*p* < 0.001). **(D)** Box plot of the cellular activity in the three different conditions, which shows the significant increase in activity in nicotine and wash conditions as compared to control (***p < 0.001, *n* = 146, six experiments). **(E)** Raster plot showing the spontaneous activity of 27 cells from a single experiment in RMTg nucleus. In the presence of MLA (100 nM, black bar), nicotine (gray band) did not increase cell activity. **(F)** The histogram shows that coactivity peaks did not change in the different conditions. **(G)** Cumulative distributions show no differences of cellular activity among the three conditions (*p* > 0.05). **(H)** Box plot of the cellular activity in the three different conditions did not show statistically significant changes of cell activity in nicotine in the presence of MLA (MLA+Nic) or wash conditions as compared to control (*p* > 0.05, *n* = 143, five experiments).

We performed calcium imaging experiments to explore the effect of nicotine on the activity of multiple cells simultaneously in the RMTg nucleus. A schematic drawing of a coronal slice is shown in Figure 8A (left). The zone within the dashed square was amplified (right), and the gray rectangles show the area within the RMTg nucleus where image recordings were made. Recordings were made in the left or the right RMTg indistinctly. Figure 8B shows calcium imaging recordings in control (left) and nicotine (right) conditions from a single experiment. Each image was obtained by taking a single frame from the video. A circular template was used to mark off each active cell and construct the coordinates map as shown in Figure 8C. The red arrows indicate the regions corresponding to active cells whose fluorescence increased with nicotine. Figure 8D shows the calcium transients (top) and their first derivative (bottom) corresponding to the encircled neuron in Figure 8B (right, red circle). Calcium transients were obtained from all the active cells recorded during the experiment in the different conditions, and their first derivatives were used to make the binary matrix, which in turn was used to construct the raster plots (see section “Materials and Methods”). Figure 9A shows a raster plot that illustrates the activity of 36 cells from a single experiment. Each row represents the activity of a cell as a function of time. The dots represent the activity of a given neuron in a frame. The raster plot illustrates three different conditions: control, nicotine (1 μM, gray band), and wash (Figure 9A). After nicotine, there is an increase in the activity that persists during the washout. The histogram in the bottom shows peaks of coactive neurons that appear after nicotine addition and increase during wash time (Figure 9B). Figures 9C,D illustrate the summary of six experiments (146 neurons). The cumulative distribution of the cellular activity (Figure 9C) shows that nicotine shifted the curve (red line) to the right and remained displaced during wash conditions (blue line). Nicotine and wash distributions were statistically different to control (black line) (Kolmogorov–Smirnov test, *p* = 0.001). The box plot (Figure 9D) also illustrates that there is a significant increase in cellular activity in nicotine and washout conditions as compared to control (Friedman test, *p* = 0.001). The raster plot in Figure 9E represents 27 cells from a single experiment. It shows that MLA (black bar) blocked the increase in the cell activity induced by nicotine (gray band). MLA by itself did not have any effect on the cell activity. Synchrony peaks of activity were not observed in any of the three conditions: control, nicotine, or wash (Figure 9F). Figures 9G,H illustrate the summary of five experiments (143 neurons). The cumulative distribution of the cellular activity (Figure 9G) shows that there were no differences between the three conditions (Kolmogorov–Smirnov test, *p* = 0.1632). The box plot did not show statistically significant changes in cell activity either (Figure 9H) (Friedman test, *p* = 0.1521).

## Discussion

The electrophysiological data presented in this study argue that nicotine promotes a long-lasting enhancement of glutamate release in the RMTg nucleus. This was measured as an increase in the frequency and amplitude of sEPSCs recorded from identified GABAergic neurons. Nicotinic effects were mediated by the activation α7 but not α4β2 nAChRs. Furthermore, the α7 nAChR selective agonist PNU-282987 mimicked the effects of nicotine. We found variations in the time course and the delay of the effect of nicotine in different cells. We noticed that recorded cells close to the surface of the slice responded faster than that located at deeper levels. Therefore, it is possible that variations of the diffusion time for the drug to reach the cells recorded could explain the delay of the effects. With respect to the time course, the variations could depend on the proportion and integrity of the glutamatergic terminals preserved in the slice and each cell recorded. However, we observed that nicotine produced long-lasting effects after the drug washout.

### Nicotinic Actions Are Presynaptic

Nicotinic actions were presynaptic as buffering intracellular calcium BAPTA, inside the recorded cells, did not prevent the effects. In contrast, BAPTA-AM, which is capable of entering the somas and terminals, suppressed the effect of nicotine. Besides, the local application of ACh on RMTg neurons did not evoke any response (Figure 5D), which discards any postsynaptic effects. This also indicates that nicotine-induced glutamate release depends on an increase in calcium levels in the axon terminals. Eserine, an acetylcholinesterase inhibitor, also mimicked the effects of nicotine suggesting the presence of an endogenous cholinergic tone that regulates glutamate release and basal excitability in the RMTg nucleus. Our results agree with those obtained in a previous study where it was reported that nicotine increases the amplitude of excitatory postsynaptic currents in the RMTg, evoked by electrical stimulation (Lecca et al., 2011).

### Nicotine Effects Depend on Intracellular Calcium

**FIGURE 10:**
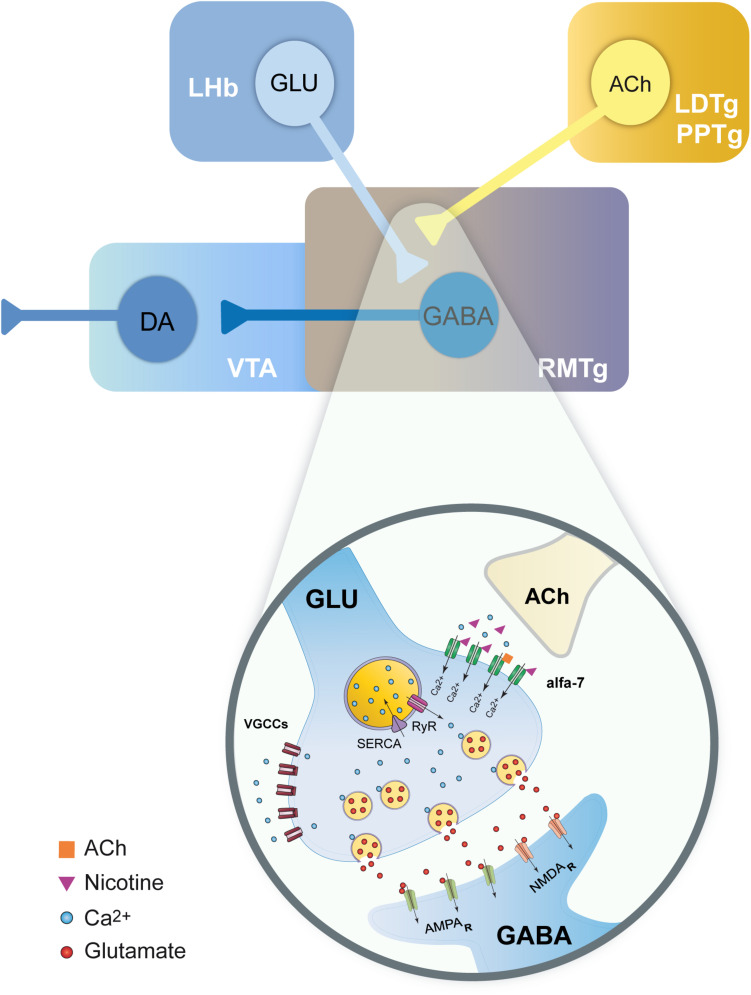
Model summarizing the effects of nicotine on glutamate terminals in the RMTg nucleus. Scheme illustrating the main inputs to the RMTg and the neurotransmitters involved (top). RMTg nucleus receives glutamate (GLU) inputs from the lateral habenula (LHb) and cholinergic (ACh) inputs from the laterodorsal (LDTg) and pedunculopontine (PPTg) tegmental nuclei. RMTg exerts a GABA-mediated inhibitory effect on the dopaminergic (DA) cells of the ventral tegmental area (VTA). A detailed view of the action mechanisms of nicotine on the LHb glutamate terminals is shown (bottom). Nicotine activates α7 nAChRs. The calcium influx through these nicotinic receptors will, in turn, evoke CICR from the endoplasmic reticulum (ER) through the activation of ryanodine receptors (RyR). This will result in long-lasting synchronized release of glutamate and an increased excitability of the RMTg GABAergic neurons.

Our data showed that nicotine effects are dependent on an increase in intracellular calcium and a CICR mechanism as CPA and thapsigargin, two blockers of SERCA, abolished nicotine-induced enhancement of sEPSCs. In contrast, cadmium did not block nicotine-induced sEPSC enhancement indicating that the activation of VGCCs is not a necessary condition for these effects. Because α7 nAChRs are 10 times more calcium-permeable than α4β2 nAChRs (Role and Berg, 1996), calcium influx through presynaptic α7 nAChRs is enough to induce CICR and neurotransmitter release without the intervention of VGCCs (Figure 10; Gray et al., 1996; Sharma and Vijayaraghavan, 2003; Sharma et al., 2008). On the other hand, the neurotransmitter release exerted through β2 subunit–containing nAChRs is entirely dependent on the activation of VGCCs, which induces an additional increase in intracellular calcium levels and CICR (Tsuneki et al., 2000; Shoop et al., 2001; Dajas-Bailador et al., 2002; Dickinson et al., 2008; Garduño et al., 2012).

### Nicotine Caused Synchronic Release From Glutamate Terminals

In most of the cells recorded, nicotine not only increased the frequency and the amplitude but also evoked bursts of EPSCs. Substitution of 90% of extracellular calcium with the divalent cations, strontium, or barium abolished the bursting and decreased the nicotine-induced amplitude enhancement of sEPSCs. Divalent cations also produced a small but significant decrease in sEPSC frequency, which was previously enhanced by nicotine (Figure 7). These data suggest that nicotine is causing a synchronic glutamate release from axon terminals by a calcium-dependent mechanism (Sharma and Vijayaraghavan, 2003; Sharma et al., 2008). This synchronic release is disrupted by replacing the calcium with the divalent cations (Léna and Changeux, 1997; Good and Lupica, 2009). Our data also agree with previous works reporting that nicotinic actions through α7 and α4β2 nAChRs involve a CICR mechanism that increases release synchrony and synaptic efficiency in different brain areas (Dajas-Bailador et al., 2002; Dickinson et al., 2008; Sharma et al., 2008; Garduño et al., 2012). In support of these findings, it was reported that presynaptic calcium stores regulate neurotransmitter release in different brain areas (Llano et al., 2000; Conti et al., 2004).

### Nicotine Caused Long-Lasting Excitatory Effects

Our results also showed that nicotinic effects were persistent for about 20–40 min after drug washout and that these effects were independent of action potential as TTX did not prevent the nicotine-induced increase in frequency or amplitude of mEPSCs. The increasing effect on the amplitude lasted longer than that of the frequency (Figure 1C) suggesting that synaptic release remained potentiated long after the drug washout. Calcium imaging experiments are consistent with these results because nicotine increased the excitability of RMTg nucleus neurons, and the effect persisted more than 20 min after nicotine was removed from the bath solution. Moreover, synchrony peaks indicating coactive neurons were more frequent during the wash time (Figure 9A).

### Physiological Relevance

In the present study, we used nicotine at a concentration of 1 μM, which is physiologically relevant. This concentration is in the range of that found in the plasma of smokers after smoking a cigarette (Dani and Heinemann, 1996). This low nicotine concentration was able to produce a long-lasting synchronized release of glutamate in the RMTg nucleus. Nicotine caused long-term synaptic changes in a way that was independent of action potentials.

### Nicotinic Increase in Glutamate Release Persists Beyond Desensitization of nAChRs

It is well known that nicotine produces rapid desensitization of nAChRs (Wooltorton et al., 2003). However, the increase in synaptic efficiency persisted a long time after desensitization occurred. Calcium influx through α7 nAChRs would cause CICR (Figure 10) and activation of calcium-dependent kinases (Dickinson et al., 2008; Sharma et al., 2008; Garduño et al., 2012; Zhong et al., 2013), resulting in this form of long-term synaptic plasticity. This provoked sustained excitability and peaks of coactive neurons as shown in calcium imaging experiments. Therefore, nicotine alters network activity by facilitating the synchronic activity of RMTg neurons.

### Nicotine Can Produce Rewarding and Aversive Effects

It is well known that nicotine activates nAChRs on diverse reward areas such as the VTA (Mansvelder et al., 2002; Miller and Picciotto, 2016) and the accumbens nucleus (Pontieri et al., 1996; Picciotto et al., 1998; Zoli et al., 2002). Nicotinic stimulation of reward structures is considered to play a key role in the establishment and maintenance of the tobacco habit (Kenny and Markou, 2006). However, nicotine also produces aversive effects mediated by several brain structures including the medial habenula (MHb) and the IPN, among others (Fowler et al., 2013; Shih et al., 2014) (see Fowler and Kenny, 2014, for review). Recent studies showed that the activation of the MHb-IPN circuit abolishes nicotine-evoked reward and decreases nicotine intake (Tuesta et al., 2017). Also, it was proposed that nicotine mediates aversive effects by modulating GABAergic IPN inputs to the LDTg nucleus and regulating the LDTg-VTA pathway (Wolfman et al., 2018).

### The RMTg Nucleus Mediates the Aversive Effects of Nicotine

According to our data, the actions of nicotine in the RMTg nucleus would also produce aversive effects as an increase in GABAergic neurons activity would inhibit the mesolimbic reward systems. In support of this idea, it was reported that the stimulation of the glutamatergic inputs to the RMTg evokes aversion to stimuli that are normally innocuous (Stamatakis and Stuber, 2012). Furthermore, it was shown that RMTg neurons projecting to the VTA encode negative but not positive motivational stimuli (Li et al., 2019a,b). Increasing the excitability of the circuits mediating the noxious properties of nicotine could be an effective strategy to decrease nicotine addiction (Fowler and Kenny, 2014). Accordingly, it was observed that aversive reactions to nicotine are important to decrease the probability of developing habitual tobacco use in first-time smokers (Sartor et al., 2010). Therefore, the RMTg nucleus could be a potential therapeutic target against tobacco addiction. For instance, the use of a positive allosteric modulator of α7 nAChRs would potentiate the noxious effects of nicotine by increasing the synchronic firing of RMTg neurons and inhibiting the VTA dopaminergic system.

## Data Availability Statement

The raw data supporting the conclusions of this article will be made available by the authors, without undue reservation, to any qualified researcher.

## Ethics Statement

The animal study was reviewed and approved by the Comité Interno para el Cuidado y Uso de Animales de Laboratorio (CICUAL), Facultad de Medicina UNAM.

## Author Contributions

DC-R performed the experiments, analyzed the data, and wrote the manuscript. ER-S performed the experiments and analyzed the data. GA-L performed immunocytochemical work. JG performed immunocytochemical work and revised the manuscript. OH-G analyzed the data. SM analyzed the data and revised the manuscript. SH-L designed the experiments, prepared the figures, and wrote the manuscript. All authors contributed to the article and approved the submitted version.

## Conflict of Interest

The authors declare that the research was conducted in the absence of any commercial or financial relationships that could be construed as a potential conflict of interest.

## Supplementary Material

Supplementary Figure 1 Time-amplitude histogram showing the time course of nicotine effect in the presence of TTX. The graph in black shows the time course of the mEPSCs frequency. For clarity, the errors were removed from the graph.

Supplementary Figure 2 Time-amplitude histograms showing the time course of nicotine effect in the presence of MLA **(A)** and DHβE **(B)**. The graphs in black show the time course of the sEPSCs frequency in both graphs. For clarity, the errors were removed from the graphs.

Supplementary Figure 3 **(A)** Time-amplitude histogram showing the time course of the effect of PNU-282987, a selective agonist of α7 nAChRs. The graph in black shows the time course of the sEPSCs frequency. For clarity, the errors were removed from the graph. **(B)** Time-amplitude histogram showing the time course of the effect of RJR, a selective agonist of the α4β2 nAChRs. The graph in black shows the time course of the sEPSCs frequency. For clarity, the errors were removed from the graph.

Supplementary Figure 4 **(A)** Time-amplitude histogram showing the time course of the nicotine effect with BAPTA in the recording pipette. The graph in black show the time course of the sEPSCs frequency. For clarity, the errors were removed from the graph. **(B)** Time-amplitude histogram showing the temporal course of the nicotine’s effect in the presence of the membrane permeable BAPTA-AM added in the bath solution. The graph in black show the time course of the sEPSCs frequency. For clarity, the errors were removed from the graph.

Supplementary Figure 5 **(A)** Time-amplitude histogram showing the time course of the nicotine effect in the presence of thapsigargin. The graph in black show the time course of the sEPSCs frequency. For clarity, the errors were removed from the graph. **(B)** Time-amplitude histogram showing the time course of the nicotine effect in the presence of CPA. The graph in black show the time course of the sEPSCs frequency. For clarity, the errors were removed from the graph. **(C)** Time-amplitude histogram showing the time course of the nicotine effect in the presence of cadmium. The graph in black, show the time course of the sEPSCs frequency. For clarity, the errors were removed from the graph.
