# Supplementary figures and images for: Nicotine Increases Spontaneous Glutamate Release in the Rostromedial Tegmental Nucleus
Source: Front Neurosci. 2021 Jan 13;14:604583. doi: 10.3389/fnins.2020.604583 (PMC7838497; doi:10.3389/fnins.2020.604583)

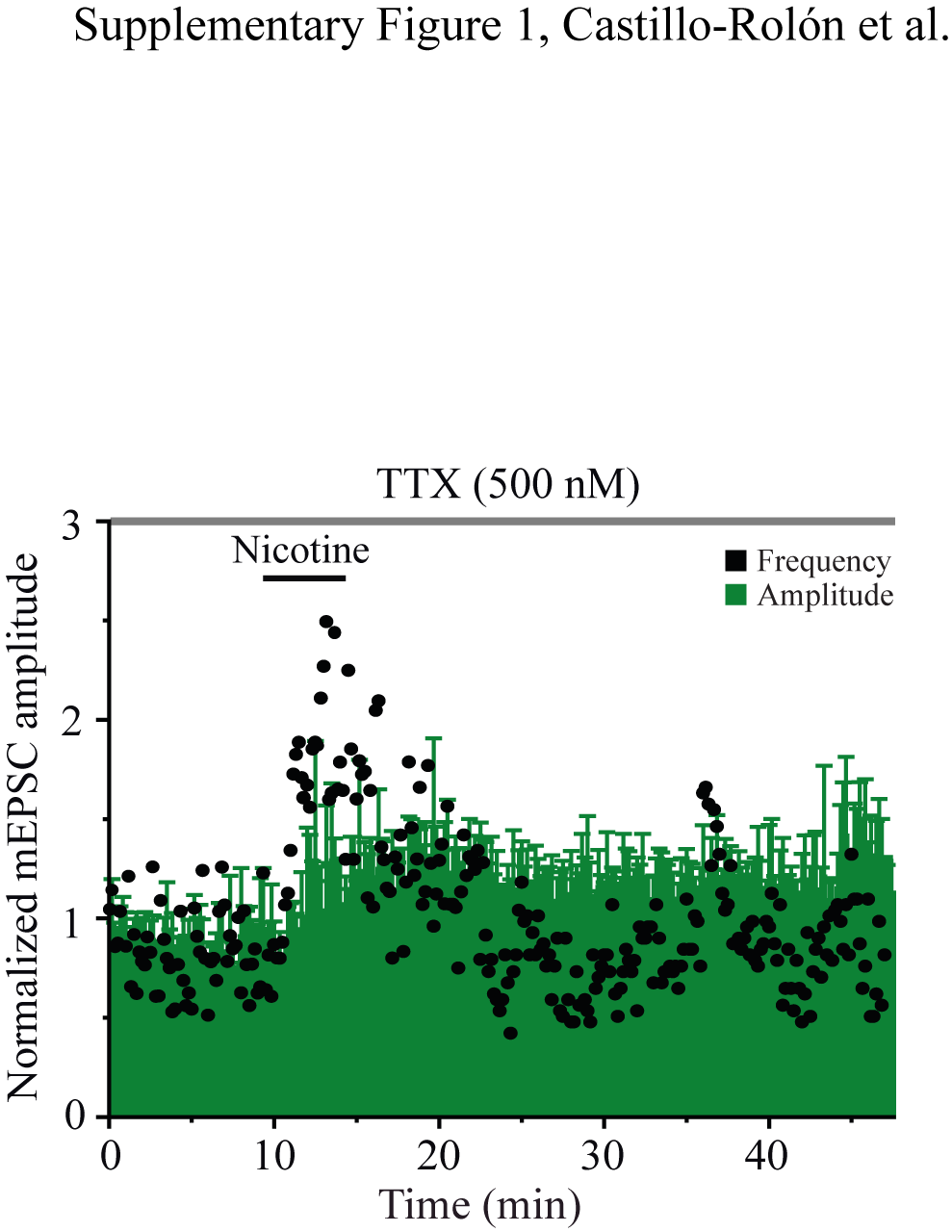

Supplement: Supplementary Figure 1 — Time-amplitude histogram showing the time course of nicotine effect in the presence of TTX. The graph in black shows the time course of the mEPSCs frequency. For clarity, the errors were removed from the graph. [file Image_1.TIF]

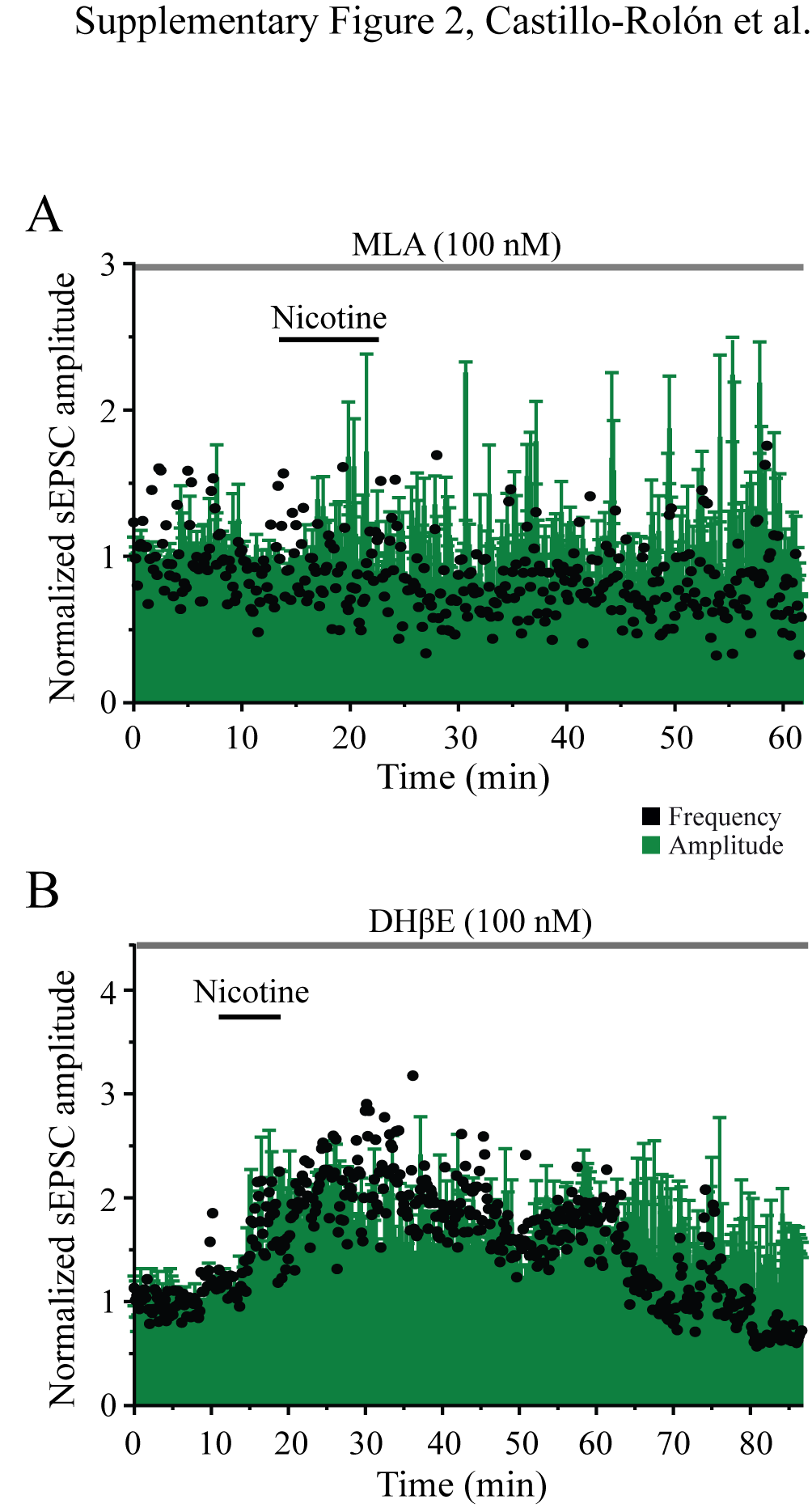

Supplement: Supplementary Figure 2 — Time-amplitude histograms showing the time course of nicotine effect in the presence of MLA (A) and DHβE (B). The graphs in black show the time course of the sEPSCs frequency in both graphs. For clarity, the errors were removed from the graphs. [file Image_2.TIF]

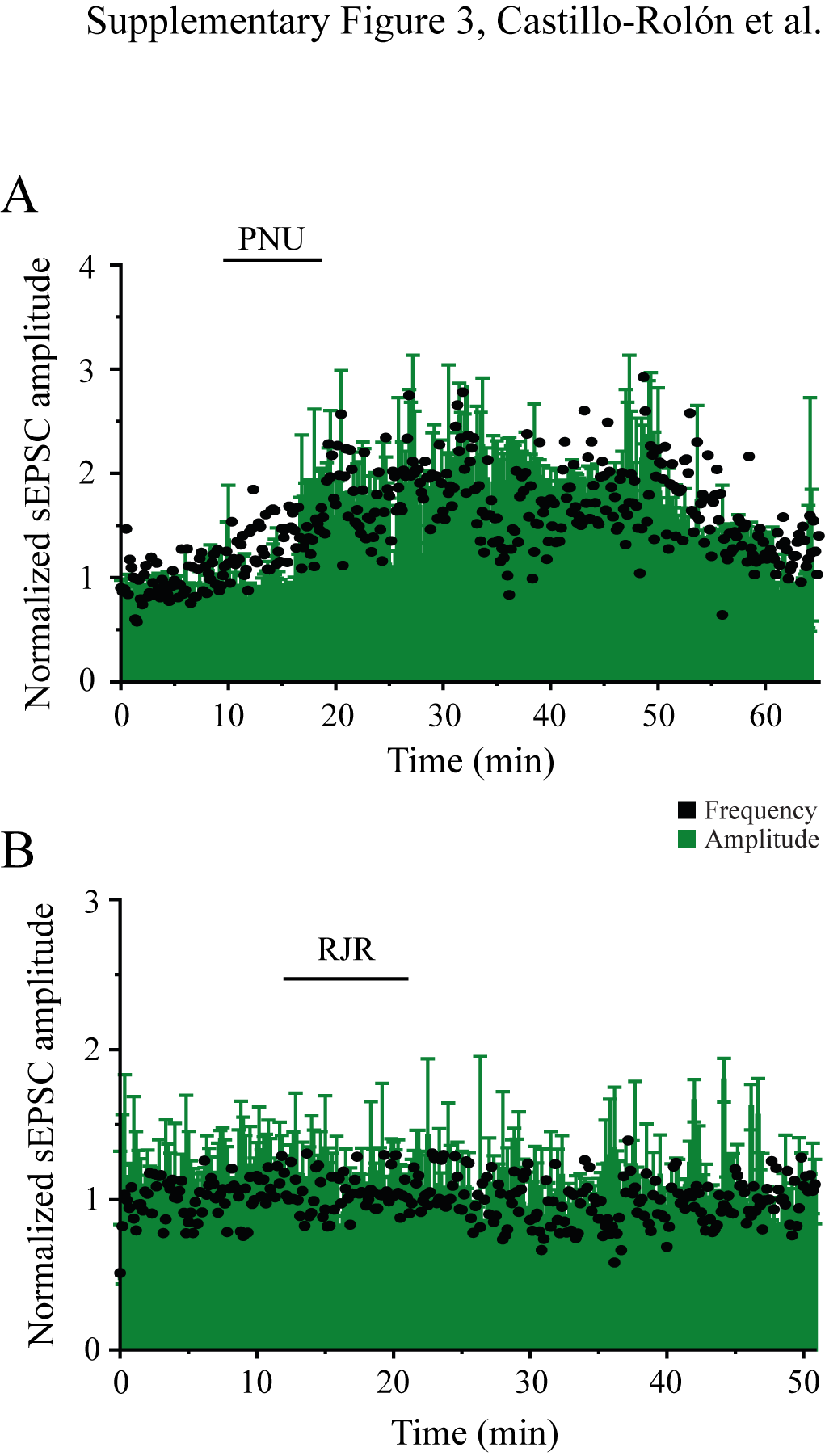

Supplement: Supplementary Figure 3 — (A) Time-amplitude histogram showing the time course of the effect of PNU-282987, a selective agonist of α7 nAChRs. The graph in black shows the time course of the sEPSCs frequency. For clarity, the errors were removed from the graph. (B) Time-amplitude histogram showing the time course of the effect of RJR, a selective agonist of the α4β2 nAChRs. The graph in black shows the time course of the sEPSCs frequency. For clarity, the errors were removed from the graph. [file Image_3.TIF]

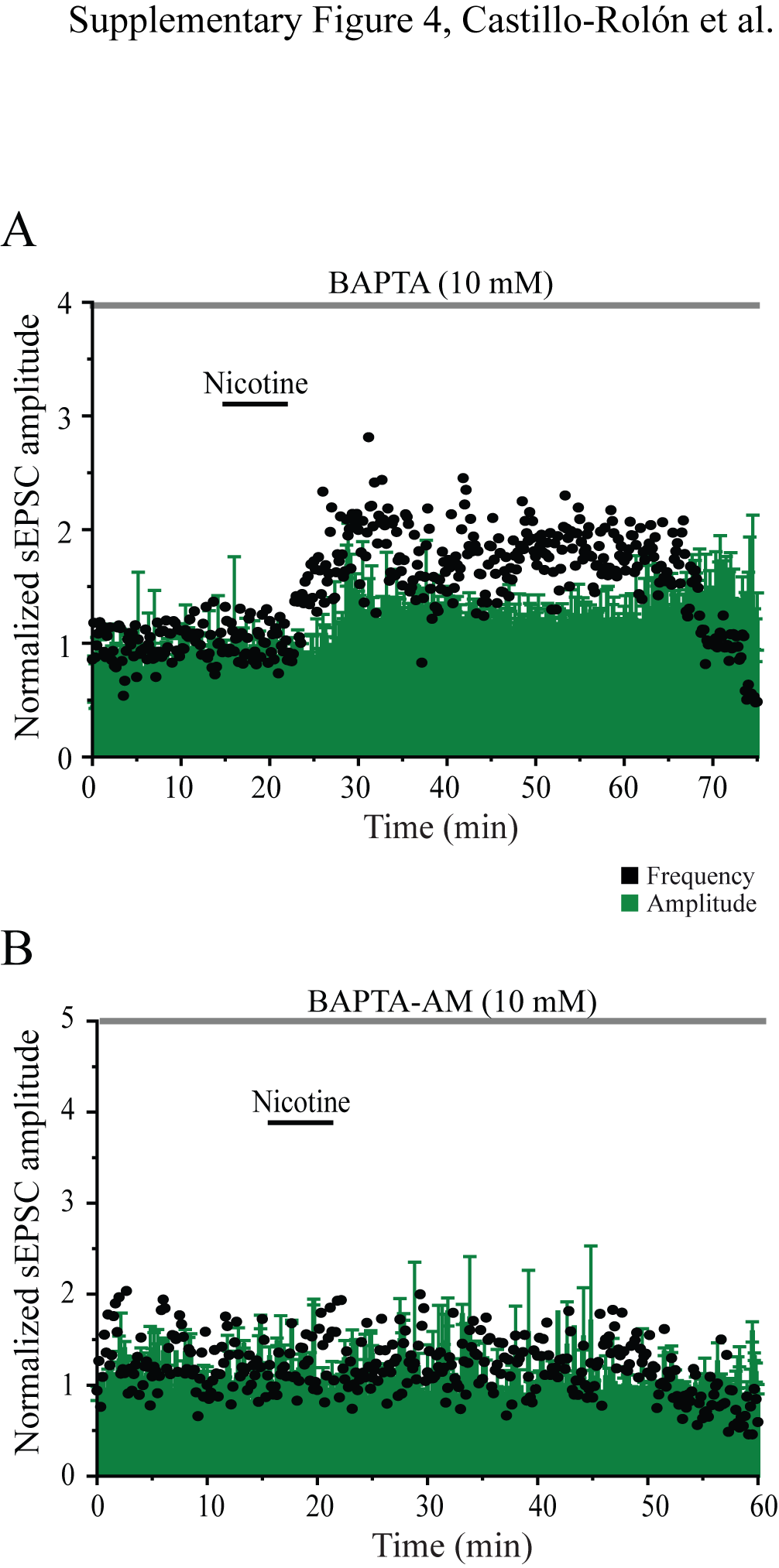

Supplement: Supplementary Figure 4 — (A) Time-amplitude histogram showing the time course of the nicotine effect with BAPTA in the recording pipette. The graph in black show the time course of the sEPSCs frequency. For clarity, the errors were removed from the graph. (B) Time-amplitude histogram showing the temporal course of the nicotine’s effect in the presence of the membrane permeable BAPTA-AM added in the bath solution. The graph in black show the time course of the sEPSCs frequency. For clarity, the errors were removed from the graph. [file Image_4.TIF]

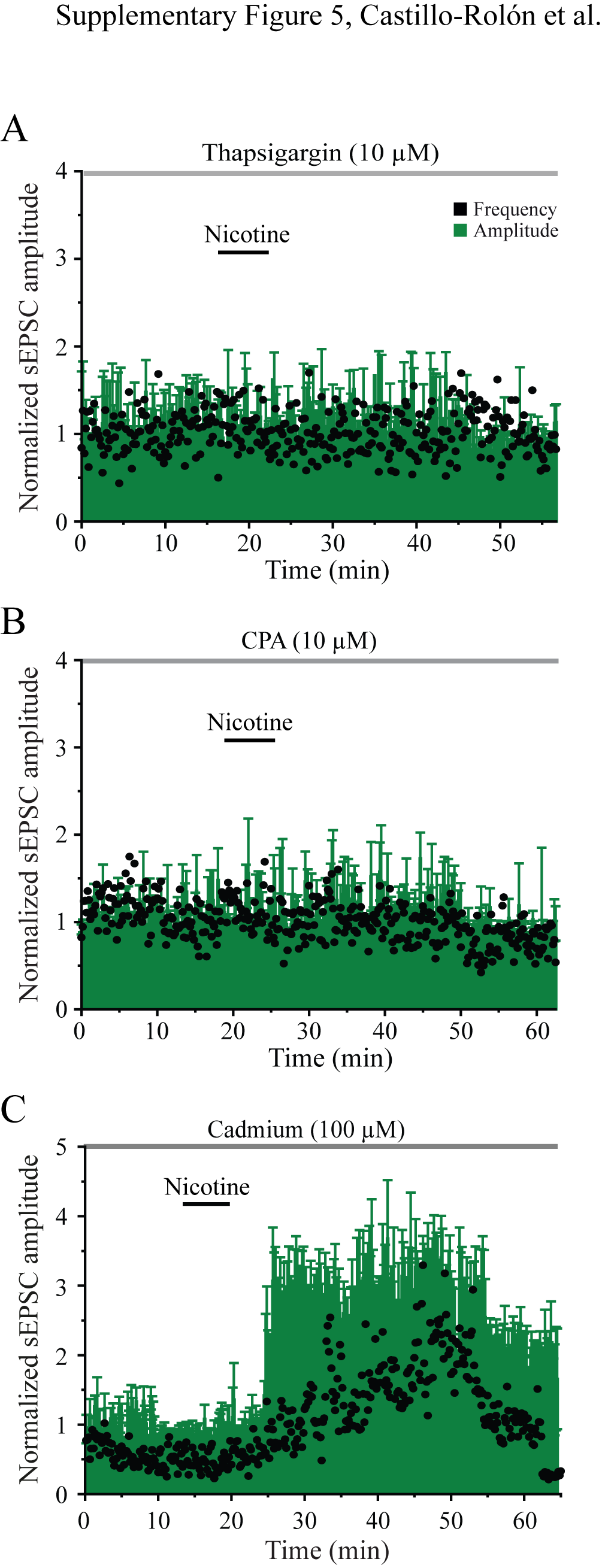

Supplement: Supplementary Figure 5 — (A) Time-amplitude histogram showing the time course of the nicotine effect in the presence of thapsigargin. The graph in black show the time course of the sEPSCs frequency. For clarity, the errors were removed from the graph. (B) Time-amplitude histogram showing the time course of the nicotine effect in the presence of CPA. The graph in black show the time course of the sEPSCs frequency. For clarity, the errors were removed from the graph. (C) Time-amplitude histogram showing the time course of the nicotine effect in the presence of cadmium. The graph in black, show the time course of the sEPSCs frequency. For clarity, the errors were removed from the graph. [file Image_5.TIF]
